# The *Nanos3*-3′UTR Is Required for Germ Cell Specific NANOS3 Expression in Mouse Embryos

**DOI:** 10.1371/journal.pone.0009300

**Published:** 2010-02-18

**Authors:** Hitomi Suzuki, Rie Saba, Aiko Sada, Yumiko Saga

**Affiliations:** 1 Department of Biological Sciences, Graduate School of Science, University of Tokyo, Tokyo, Japan; 2 Division of Mammalian Development, National Institute of Genetics, Shizuoka, Japan; 3 Department of Genetics, The Graduate University for Advanced Studies (Sokendai), Shizuoka, Japan; Victor Chang Cardiac Research Institute (VCCRI), Australia

## Abstract

**Background:**

The regulation of gene expression via a 3′ untranslated region (UTR) plays essential roles in the discrimination of the germ cell lineage from somatic cells during embryogenesis. This is fundamental to the continuation of a species. Mouse NANOS3 is an essential protein required for the germ cell maintenance and is specifically expressed in these cells. However, the regulatory mechanisms that restrict the expression of this gene in the germ cells is largely unknown at present.

**Methodology/Principal Findings:**

In our current study, we show that differences in the stability of *Nanos3* mRNA between germ cells and somatic cells is brought about in a 3′UTR-dependent manner in mouse embryos. Although *Nanos3* is transcribed in both cell lineages, it is efficiently translated only in the germ lineage. We also find that the translational suppression of NANOS3 in somatic cells is caused by a 3′UTR-mediated mRNA destabilizing mechanism. Surprisingly, even when under the control of the CAG promoter which induces strong ubiquitous transcription in both germ cells and somatic cells, the addition of the *Nanos3*-3′UTR sequence to the coding region of exogenous gene was effective in restricting protein expression in germ cells.

**Conclusions/Significance:**

Our current study thus suggests that *Nanos3*-3′UTR has an essential role in translational control in the mouse embryo.

## Introduction

The manner in which genes are regulated to produce the correct combination of proteins for every cell type remains a fundamental question in biology. In many cases, gene expression is primarily regulated via transcription under the control of enhancer and promoter sequences. However, it is now becoming clear that post-transcriptional regulation mediated via a 3′ untranslated region (UTR) plays key roles in the control of mRNA stability and/or translation. A critical step in the establishment of elaborate germ cell lineages during early embryogenesis in nematodes, fly, fish and frog is the temporal and spatial regulation of several proteins via mechanisms that are dependent on the 3′UTR of maternal mRNAs including *nanos* [1], [2], [3], [4].

The *nanos* genes are evolutionarily conserved among many organisms and play important roles during germ cell development [5], [6], [7], [8], [9], [10], [11]. During germ cell specification in *Drosophila*, maternal *nanos* mRNA becomes localized in the germ plasm in the posterior part of the egg [12], [13]. This localization is inefficient, however, and translational repression is therefore essential for the restricted production of Nanos protein in the posterior region. This repression is mediated by a 90-nucleotide translational control element (TCE) in the 3′UTR of *nanos* mRNA [14], [15], [16], [17], [18] to which Smaug (Smg) or Glorund (Glo) bind [19], [20], [21]. On the other hand, the localization in the germ plasm and subsequent translational activation of *nanos* mRNA is regulated by the Oskar (Osk) protein via 3′UTR-dependent mechanisms [19], [22], [23]. In *Danio rerio*, maternal *nanos1* mRNA is also present in a whole oocyte, but only a portion is localized to the germ plasm and translated specifically in the PGC. The translation of the bulk of *nanos1* mRNA in somatic cells is then rapidly degraded during embryogenesis. The regulation of *nanos1* both in the PGC and somatic cells depends on three elements within the *nanos1*-3′UTR: (1) a site required for its localization to the germ plasm [24]; (2) two miR430 sites responsible for mRNA degradation in somatic cells; and (3) the binding site for the Dead end 1(Dnd1) protein that is expressed only in the PGC and protects mRNA from miR430-dependent degradation [2], [25].

In *Mus musculus*, primordial germ cells (PGCs) are induced from a population of pluripotent epiblast cells [26], [27]. Following their induction, these PGC precursors translocate to the base of the allantois by E7.25 and once formed, migrate to the endoderm (E7.5), travel through the hindgut (from E8.0), dorsal mesentery and dorsal body wall, and reach the genital ridge at around E10.5 to E11.5. Following the sex differentiation of the somatic gonads, PGCs themselves differentiate into male or female germ cells at around E12.0 [28]. Three *Nanos* homologs (*Nanos1-3*) have been identified in mice, and *Nanos2* and *Nanos3* have been implicated in germ cell development [10]. NANOS2 is specifically expressed in the mouse male germ cells after their colonization of the gonads and is essential for their development. In our previous study, we reported that the *Nanos2*-3′UTR promotes the efficient translation of this protein in the male germ cell after E13.5 via an unknown mechanism [29]. *Nanos3* is expressed in the PGCs after their formation until shortly after their settlement in the gonads (E14.5 in male, E13.5 in female), and is re-expressed after birth in the testes [10]. *Nanos3* knockout mice are thus sterile because of the loss of migrating PGCs during embryogenesis. These data suggest that NANOS3 plays an important role in the maintenance and survival of PGCs [10], [30]. However, the regulatory mechanism of NANOS3 expression and the function of the *Nanos3*-3′UTR had not been fully investigated as yet.

In our present report, we show that *Nanos3* mRNA is transcribed in both germ cells and somatic cells, although NANOS3 protein is expressed specifically in germ cells. By applying a transgenic mouse strategy, we show that the translation of NANOS3 in somatic cells is suppressed via an mRNA destabilizing mechanism mediated by the *Nanos3*-3′UTR.

## Results

### The *Nanos3*-3′UTR Is Required for Suppression of Nanos3 Expression in Somatic Cells

**Figure 1:**
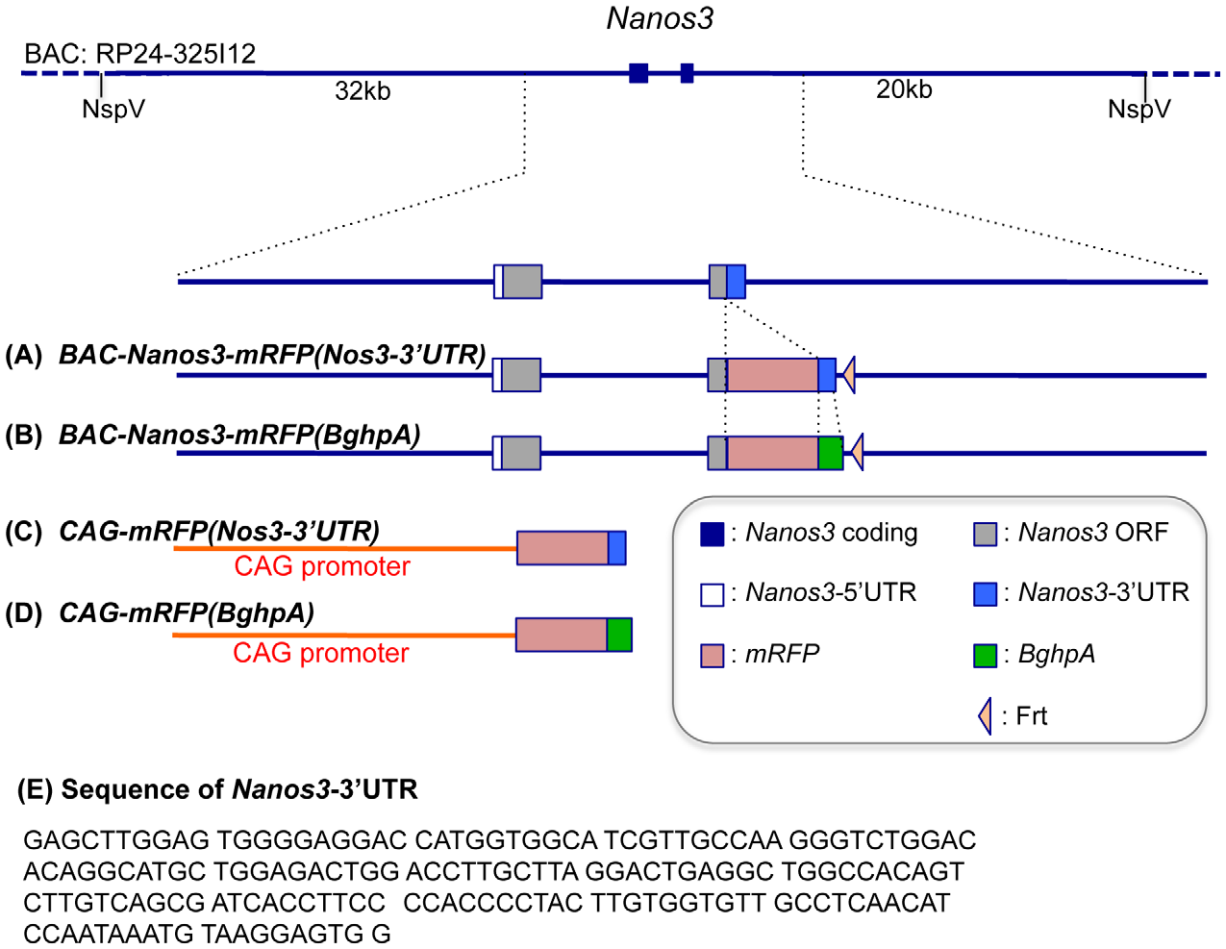
Schematic representation of the transgenes used in this study. The top line represents BAC RP24-325I12 which contains the *Nanos3* gene, the second line is a larger scale schema of a portion of this construct. (A–D) Different modifications of the transgene. Blue lines denote sequences derived from the BAC RP24-325I12 construct and the red lines those of the CAG promoter. The meanings of each box is indicated. (E) Sequence of *Nanos3*-3′UTR we used.

**Figure 2:**
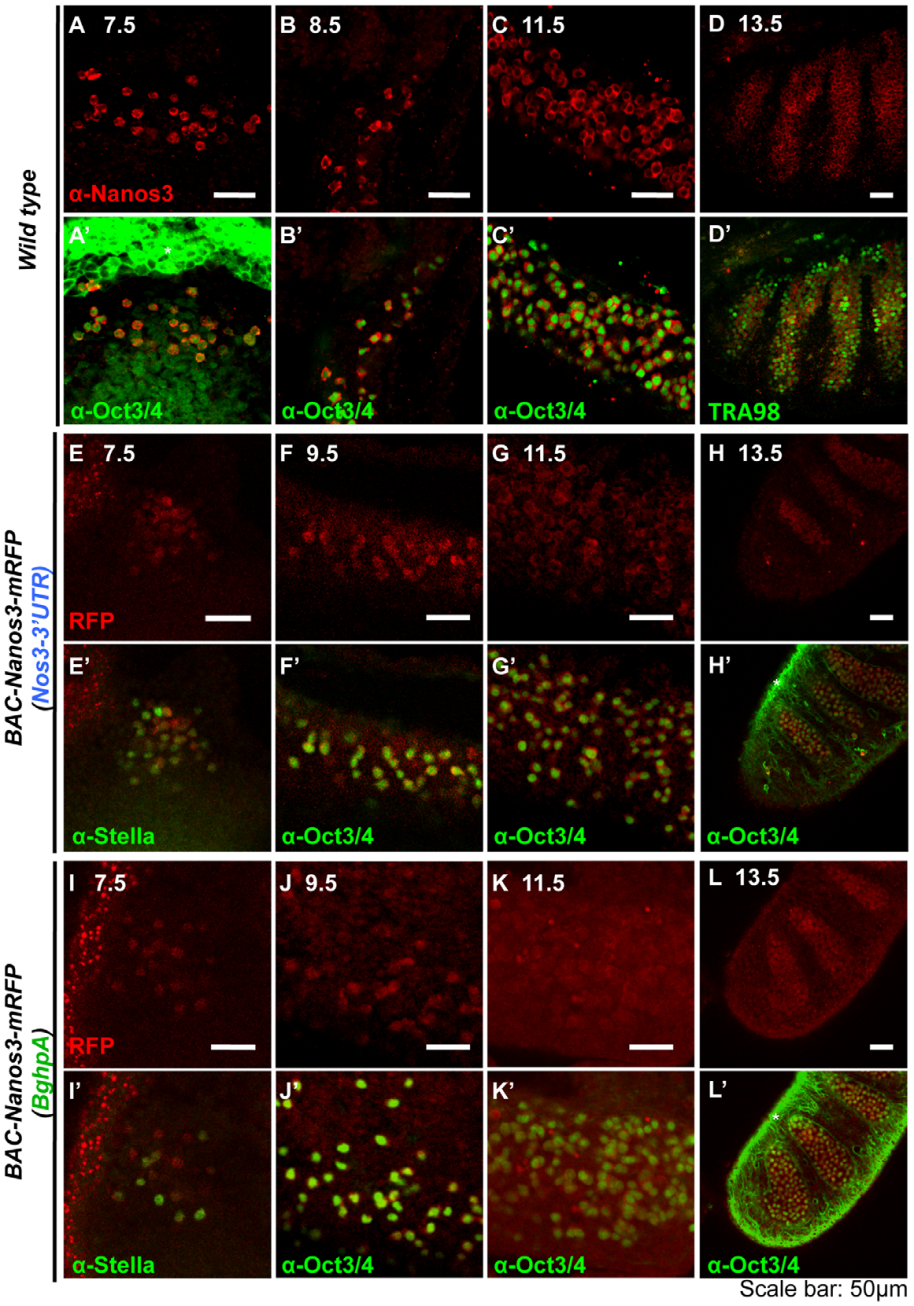
Nanos3-mRFP is expressed dominantly in germ cells in BAC transgenic mouse lines. Confocal images of embryos of the wild-type (A–D), *BAC-Nanos3-mRFP(Nos3-3′UTR)* (E-H) or *BAC-Nanos3-mRFP(BghpA)* (I–L). Panels (A–D) show images of immunostaining for anti-NANOS3. Panels (E–L) show images of mRFP fluorescence (not immunostaining) and (A′–L′) are merged images that include immunostaining for the germ cell marker anti-OCT3/4, anti-Stella/PGC7 or TRA98 (green signal). The developmental stage associated with each figure is indicated above each panel: E7.5 (A, E, and I), E8.5 (B), E9.5 (F and J), E11.5 (C, G and K), E13.5 male gonad (D, H and L). Asterisks represent non-specific signals by the secondary antibody. Scale bars, 50 µm.

To elucidate whether the *Nanos3*-3′UTR is involved in NANOS3 expression in mouse, we generated two BAC transgenic mouse lines; *BAC-Nanos3-mRFP(Nos3-3′UTR)* containing the endogenous *Nanos3*-3′UTR (Fig. 1A and E), and *BAC-Nanos3-mRFP(BghpA)* harboring an exogenous 3′UTR, *Bovine growth hormone poly(A) signal* (*BghpA*; Fig. 1B). We first confirmed that either *BAC-Nanos3-mRFP(Nos3-3′UTR)* or *BAC-Nanos3-mRFP(BghpA)* could rescue the *Nanos3^−/−^* embryonic phenotype (Fig. S1), indicating that this BAC construct contains regulatory elements that are sufficient to maintain endogenous NANOS3 expression and the NANOS3-mRFP protein was functional. The NANOS3-mRFP expression in germ cells in both transgenic embryos showed a similar pattern to the endogenous protein (Fig. 2 and S2) exhibiting cytoplasmic localization as seen for NANOS2 [31]. The small difference between Nanos3 and Nanos3-mRFP was that the former was clearly localized to the cytoplasm whereas the latter was less clear and slightly localized in nuclei also. It was consistent with a previous report [32].

**Figure 3:**
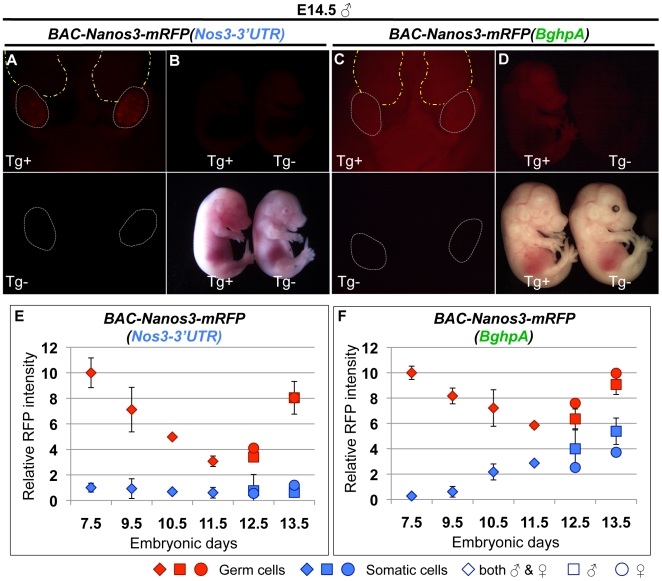
Replacement of *Nos3-*3′UTR with *BghpA* results in the upregulation of NANOS3-mRFP protein in somatic tissues. (A–D) Fluorescence images of male embryos derived from *BAC-Nanos3-mRFP(Nos3-3′UTR)* (A–B) and *BAC-Nanos3-mRFP(BghpA)* (C–D) transgenic embryos at E14.5. (A and C) The upper images are of the abdomens of embryos harboring the transgene (Tg^+^), whereas the lower images are of the same tissues from embryos with no transgene (Tg^−^). The broken gray lines indicate the gonads and broken yellow lines indicate the kidneys. (B and C) Whole body of Tg+ and Tg- embryos are shown. The image in the upper panel shows the mRFP fluorescence pattern, whilst the lower panels are the corresponding bright field images. (E and F) Developmental changes in the relative mRFP intensities in germ cells (red) and somatic cells (blue) derived from *BAC-Nanos3-mRFP(Nos3-3′UTR)* (E) and *BAC-Nanos3-mRFP(BghpA)* (F) transgenic embryos. Error bars represent the s.e.m.

Interestingly, however, in the embryo harboring *BAC-Nanos3-mRFP(BghpA),* the intensity of NANOS3-mRFP was gradually increased in the somatic tissues at later embryonic stage (Fig. 2K-L). In the E14.5 male, a striped pattern was observed for *BAC-Nanos3-mRFP(Nos3-3′UTR)* reflecting germ cell localization in the testis cords in gonads, whereas the pattern was unclear in *BAC-Nanos3-mRFP(BghpA)*, indicating strong expression in the surrounding somatic tissues (compare Figs. 2H and 2L, 3A–D). In addition, the whole body of the *BAC-Nanos3-mRFP(BghpA)* embryos expressed NANOS3-mRFP (Fig. 3D). These results suggest that *Nanos3* is transcribed in many embryonic tissues and that the *Nanos3*-3′UTR is required to suppress translation in somatic tissues.

To evaluate the suppressive effects of 3′UTR in somatic cells quantitatively, we compared the abundance of Nanos3-mRFP protein based on the intensities of mRFP signals in both the germ cells and surrounding somatic cells in each transgenic embryo from E7.5 to E13.5 using imageJ software (Fig. 3E–F). The changes in the relative mRFP intensities in the germ cells were similar between the two transgenic lines. These were gradually decreased after E7.5, reached their lowest level at E11.5 and then rapidly increased from E12.5. Contrary to the data found in the germ cells, the mRFP intensities in the surrounding somatic cells showed a clear difference between the two lines. In the embryo harboring *Nanos3-3′UTR*, the mRFP intensity was maintained at very low levels throughout embryogenesis. However, in the embryo containing the *BghpA* elements, this expression gradually increased from E10.5 and at E13.5 reached 60% of the intensity seen in the germ cells, although it was maintained at low levels at E7.5 and E9.5. These data suggest that the translation of NANOS3 is upregulated after E9.5 in somatic tissues and that the *Nanos3*-3′UTR is required to suppress this activity.

### The Accumulation of Somatic *Nanos3* mRNA Is Suppressed by the *Nanos3*-3′UTR

**Figure 4:**
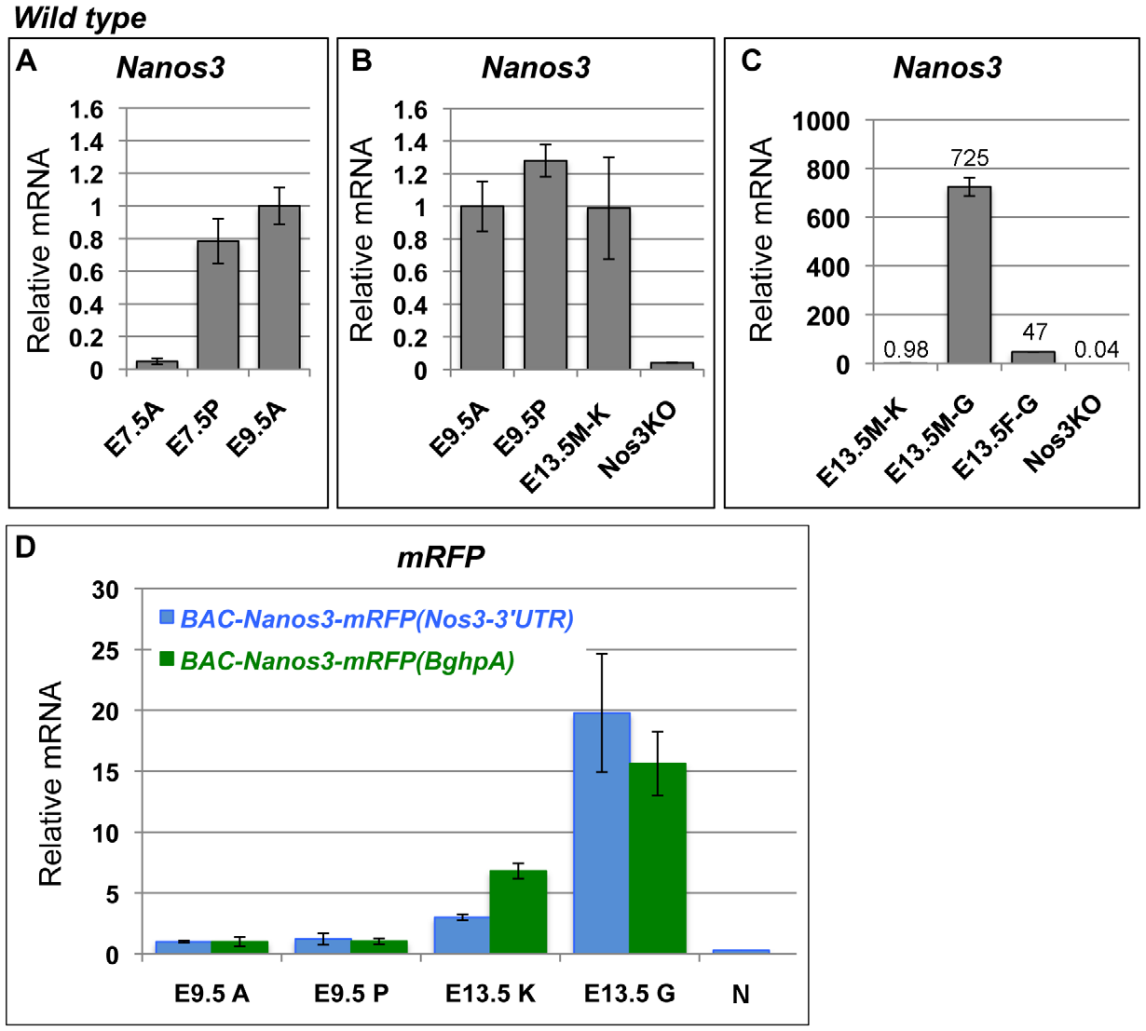
The accumulation of somatic *Nanos3* mRNA is suppressed by the *Nanos3*-3′UTR. The levels of *Nanos3* (A–C) *or mRFP* (D) mRNA were compared by quantitative RT-PCR using RNA samples derived from *wild-type* embryos (A–C) and *BAC-Nanos3-mRFP(Nos3-3′UTR)* (blue in D) and *BAC-Nanos3-mRFP(BghpA)* (green in D) transgenic embryos at E7.5, E9.5 and E13.5. Data were normalized by *G3PDH* in each sample. The relative mRNA levels in the E9.5A sample (an anterior part of E9.5 embryo) were assigned the reference value of 1.0. A, anterior part of the embryo; P, posterior part of the embryo; M–K or K, the male kidney; M–G or G, the male gonad; F–G, the female gonad; N, the posterior part of a *Nanos3* knockout embryo at E9.5. Error bars represent the s.d.

We next examined the endogenous *Nanos3* mRNA levels in somatic tissues between E9.5 and E13.5 by quantitative RT-PCR (qRT-PCR) and found transcripts even in somatic tissues (Fig. 4A–C), consistent with the above data. The anterior half of the embryo at E9.5 (9.5A) and the kidney at E13.5 (13.5K) do not contain any germ cells, but *Nanos3* mRNA was detected (Fig. 4B–C). The level of *Nanos3* mRNA in somatic tissue from E9.5 to E13.5 was maintained at very low levels compared with the gonads which containing many germ cells (Fig. 4C). Interestingly, *Nanos3* expression was not detected in the anterior half of the embryo at E7.5 (E7.5A), suggesting the transcription of this gene is restricted to the PGCs when they are formed and may be slightly increased in the somatic cells at the later stage. It is consistent with previous reports, which include single–cell PCR analyses demonstrating *Nanos3* expression exclusively in the PGCs [33] and our lineage study using Nanos3-cre [30].

In embryos harboring *BAC-Nanos3-mRFP(Nos3-3′UTR)*, the levels of *mRFP* at both stages was low, similar to *Nanos3* mRNA in the wild-type embryo (Fig. 4B and D). In contrast, in embryos harboring *BAC-Nanos3-mRFP(BghpA)*, the relative *mRFP* levels became two-fold higher at E13.5 than those of *BAC-Nanos3-mRFP(Nos3-3′UTR)* (Fig. 4D). Taken together, these data suggest that *Nanos3* is transcribed in both germ cells and somatic tissues by at least E9.5 and that *Nanos3*-3′UTR is required to suppress *Nanos3* accumulation in somatic tissues.

### The *Nanos3*-3′UTR Is Sufficient for the Establishment of Germ-Cell Specific Expression Pattern in the Mouse Embryo

**Figure 5:**
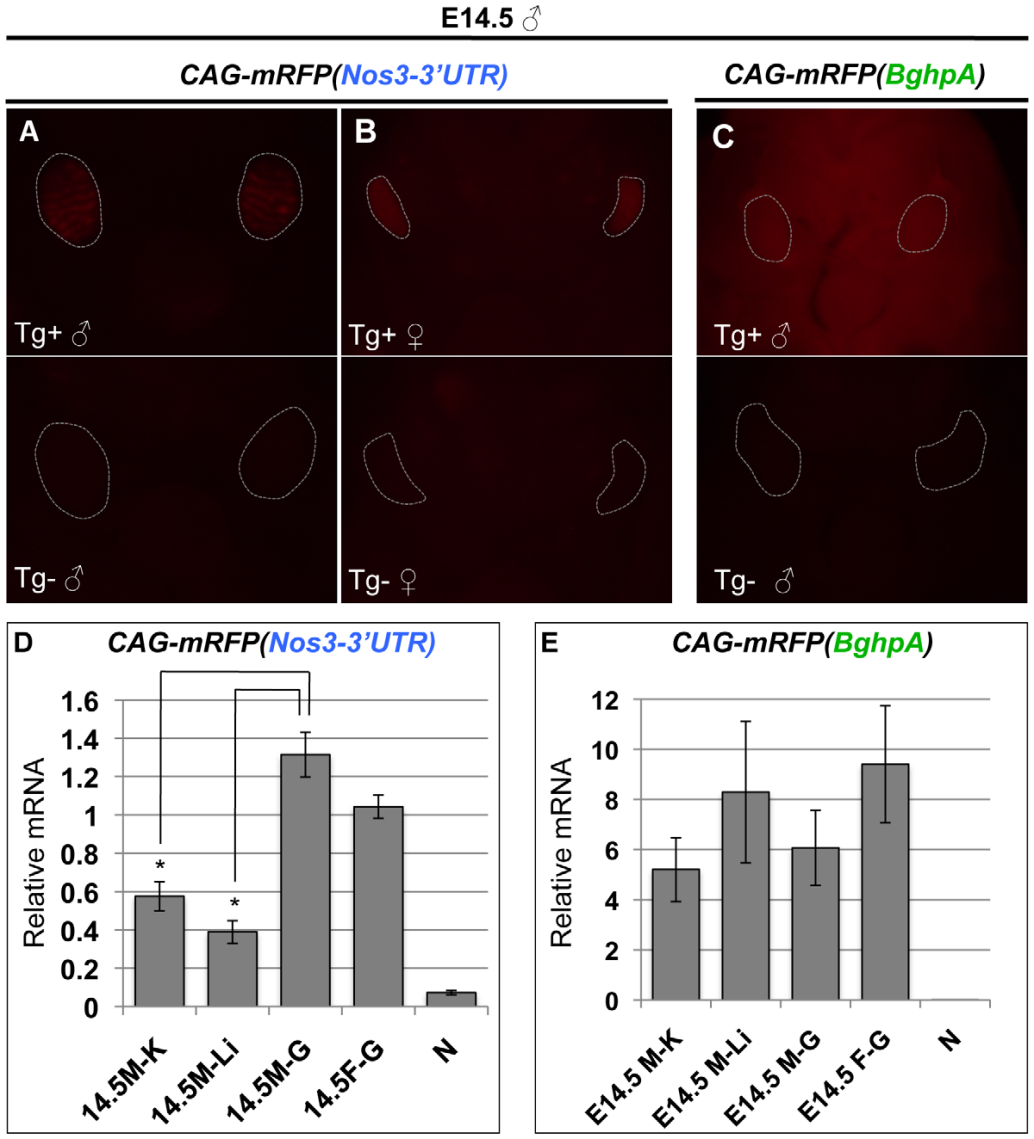
*Nanos3*-3′UTR is sufficient to establish the germ cell-specific expression pattern in the mouse embryo. (A–C) Fluorescence images of *CAG-mRFP(Nos3-3′UTR)* (A, male; B, female) and *CAG-mRFP(BghpA)* (C, male) transgenic embryos at E14.5. The upper images are of the abdomens of embryos harboring transgenes (Tg^+^), whereas the lower panels show corresponding images from embryos lacking a transgene (Tg^−^). Broken gray lines indicate gonads. (D–E) Quantitative RT-PCR analysis of *mRFP* in *CAG-mRFP(Nos3-3′UTR)* (D) or *CAG-Lyn-mRFP(BghpA)* (E) embryos at E14.5. The data were normalized using *G3PDH*. M–K, male kidney; M-Li, male limb; M–G, male gonad; F–G, female gonad; N, wild-type embryo. Error bars represent the s.d. Student t-test was used to calculate P values. *, P<0.05.

To further investigate whether the *Nanos3*-3′UTR affects the transcription or stability of mRNA, we generated two additional transgenic mice. We utilized the CAG promoter, a known strong promoter-enhancer that drives the ubiquitous transcription of mRFP with either *Nanos3*-3′UTR or *BghpA* (Fig. 1C–D). Surprisingly, *Nanos3*-3′UTR proved to be effective in restricting the mRFP expression in the germ cells at E14.5 (Fig. 5A–B), whilst mRFP was always expressed ubiquitously with no pattern observed in the *CAG-mRFP(BghpA)* embryo (Fig. 5C). The same expression pattern was observed in *CAG-lyn-mRFP(BghpA)* transgenic embryo that had been previously established in our laboratory [34]. The lyn-mRFP is an mRFP that contains the lyn kinase at its N-terminus, which serves as a membrane localization signal but does not affect neither transcription and translation [34]. Therefore, we considered *CAG-lyn-mRFP(BghpA)* is compatible with *CAG-mRFP(BghpA)*. By qRT-PCR analyses, we further revealed that the relative amounts of *mRFP* mRNA in somatic tissues (a kidney and a hind limb) were significantly lower than those in the gonads in *CAG-mRFP(Nos3-3′UTR)* embryos whereas not significantly altered in *CAG-lyn-mRFP(BghpA)* (Fig. 5D and E). The results suggest that *Nanos3*-3′UTR is sufficient to suppress protein expression in somatic cells by destabilizing mRNA and establishing a germ-cell specific expression pattern.

**Figure 6:**
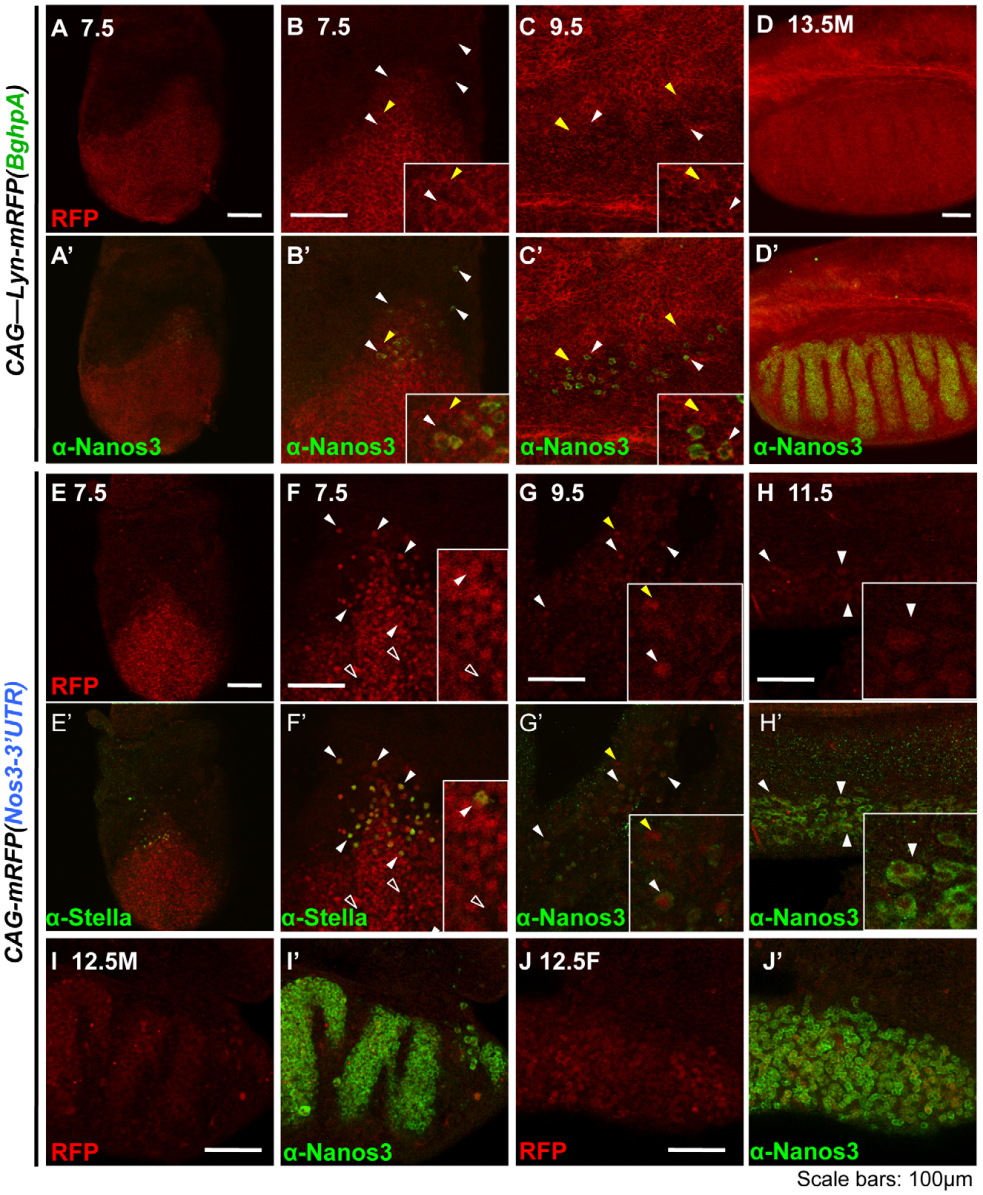
The *Nanos3*-3′UTR may function in both germ cells and somatic tissues after generation of the PGC. Confocal images of *CAG-Lyn-mRFP(BghpA)* (A–D) and *CAG-mRFP(Nos3-3′UTR)* (E–J) transgenic embryos. (A–J) mRFP fluorescence; (A′–J′) merged images of mRFP fluorescence (red) and immunostaining signals (green) for NANOS3 (A′–D′ G′–J′), STELLA/PGC7 (E′–F′). Insets are high magnification of each panel. The embryonic stage for each sample is indicated. White arrowheads, germ cells; white open arrowheads, somatic cells that do not express mRFP; yellow arrowheads, somatic cells that express mRFP. Scale bars, 100 µm.

To determine the point at which *Nanos3*-3′UTR begins to function and contribute to the establishment of the germ cell-specific pattern for NANOS3, we compared the mRFP expression patterns between *CAG-mRFP(Nos3-3′UTR)* and *CAG-lyn-mRFP(BghpA)* transgenic embryos. In *CAG-lyn-mRFP(BghpA)*, mRFP was expressed in all embryonic tissues at all stages (Fig. 6A–D). In *CAG-mRFP(Nos3-3′UTR)* however, mRFP was expressed in all embryonic cells prior to germ cell formation at E7.0, similar to the profile found in the *CAG-mRFP(BghpA)* embryo (data not shown). After PGC formation, mRFP was still found to be expressed in most embryonic cells at E7.5, but in somatic cells this expression is gradually reduced (E9.5) and the germ-cell specific pattern is almost established by E11.5 (Fig. 6E–H). At E12.5, mRFP expression in the germ cells became notably stronger than in the somatic cells of both male and female embryos (Fig. 6I–J). This germ cell specific mRFP pattern was maintained until at least E16.5 at which stage endogenous *Nanos3* expression is almost lost. These observations suggest that the *Nanos3*-3′UTR might function in all embryonic cells from E7.5 to E16.5. It is possible also that the *Nanos3-*3′UTR is involved in translational activation in germ cells. These different functions of this regulatory element in germ cells and in somatic cells might therefore contribute to the establishment of germ cell-specific NANOS3 protein expression.

It is noteworthy that the addition of the 3′UTRs of other germ cell specific genes such as *Nanos2* and *Stella/PGC7* was not sufficient to establish germ cell-specific expression patterns i.e. the transgenic embryos *CAG-mRFP(Nanos2-3′UTR)* and *CAG-mRFP(Stella-3′UTR)* showed strong and ubiquitous mRFP expression (Fig. S3). This suggests that the function of *Nanos3*-3′UTR is relatively unique in the mouse germ cell, unlike fly and nematodes in which 3′UTRs of many genes each have significant responsibility for the temporal and spatial control of a specific protein in their germlines [4], [35].

## Discussion

The regulatory mechanisms underlying gene expression remains one of the most fundamental and significant themes in biology. In our current study, we analyzed the mechanisms underlying NANOS3 expression in vivo using BAC modification and transgenic technologies. Although *Nanos3* is transcribed both in germ cells and in many somatic tissues, efficient translation of NANOS3 protein occurs only in germ cells.

It has been shown previously that the expression of maternal mRNAs depends on the corresponding 3′UTR in many animal species. The 3′UTRs of *nanos* homologs play essential roles in the respective mRNA localization, translation and degradation in *C. elegans*, *Drosophila*, and *Zebrafish* [1], [9], [36]. We have found in our present experiments that the *Nanos3*-3′UTR of *Mus musculus* also has a regulatory function during embryogenesis, even though NANOS3 is transcribed zygotically and germ cell formation in mice is quite different from other animals. There have been several reported examples of 3′UTR regulation of zygotic mRNA [37], [38], [39]. However, in all these cases, the transcripts were driven by tissue-specific promoters. Hence, *Nanos3*-3′UTR is the first example of a regulator of zygotic mRNA that can establish a tissue-specific gene expression pattern even if the mRNA is transcribed by a ubiquitous promoter.

The mechanisms of 3′UTR-dependent *nanos* mRNA regulation have been addressed previously in fishes and flies, in which miR430 and the Dnd1 protein, or the Smg, Glo and Osk proteins are involved in mRNA regulation via the *nanos*-3′UTR. In mice, one ortholog of *Dnd1* and two orthologs of *Smg* have now been identified [40], [41]. We examined the possible effects of these proteins on the translation of an mRNA harboring the *Nanos3*-3′UTR by a luciferase assay in the NIH3T3 cell line. The stability of luciferase mRNA is also affected by *Nanos3*-3′UTR. However, the addition of both proteins did not result in any effects on reporter activities (data not shown). It is possible that they need co-factors which are not expressed in this cell line. It is also possible that the abundant expression of endogenous *Smg* in NIH3T3 cells caused no effect. In addition, the sequence of *Nanos3-*3′UTR has almost no similarity to the 3′UTRs of *nanos* orthologs and has no significant match with any miRNA target sites. Although several stem-loop structures have been predicted using the ‘mfold’ program (Zuker, 2003), these are not similar to the *Drosophila* TCE (data not shown). Hence, the mechanism of *Nanos3-*3′UTR dependent regulation is still unclear and is an essential project for a future study.

The somatic expression of *BAC-Nanos3-mRFP(BghpA)* did not affect mouse development unlike in the case of fly and the biological significance of *Nanos3*-3′UTR-dependent regulation also remains unclear. Since the regulation of gene transcription appears not always to be strict, *Nanos3*-3′UTR may prevent the accumulation of waste materials in the cell by promoting mRNA degradation.

## Materials and Methods

### Mice

The methods used to generate *Nanos3-L-3′UTR* (*Nanos3^+/−^*) mice and their subsequent characterization has been previously described [10]. All mice were an MCH background (closed colony derived from an ICR strain, CREA, Japan).

### Generation of Nanos3-BAC Transgenic Mice

A *Nanos3*-BAC clone, RP24-325I12 (Invitrogen) was used for modification via the λ red recombination method as described previously [42], [43]. The vectors were constructed as follows: the *Nanos3*-3′UTR or *BghpA* sequence was inserted into the pBSIIKS vector harboring the *mRFP* gene (kindly provided by Dr. Roger Tsien [44]) and a cassette containing the *kanamycin* resistance gene flanked by two FRT sequences. The primers used for the BAC modifications are as follows (primer sequences are listed in Methods S1):

*BAC-Nanos3/mRFP-F* and *BAC-Nanos3(fusion-3′UTR)-R*

for *BAC-Nanos3-mRFP(Nos3-3′UTR), and BAC-Nanos3-mRFP(BghpA)*;

*BAC-Nanos3(ATG)/mRFP-F* and *BAC-Nanos3(fusion-3′UTR)-R*

for *BAC-ΔNos3-mRFP(Nos3-3′UTR)* and *BAC-ΔNos3-mRFP(BghpA)*. The primers used to confirm recombination were *N3-IN-F1* and *N3-LA-KR1*. All PCR reactions were performed using Prime STAR DNA polymerase (Takara).

### Cloning of 3′UTR Sequences

The *Nanos3-*3′UTR was cloned by PCR using a DNA template prepared from the tail of a C57BL6/J mouse. The primers used were *N3-stop-SalI-F1* and *N3-3′U-HindIII-R1*.

### Generation of Transgenic Mice

All BAC constructs were digested with Csp45I and PmacI (Takara) and then gel purified. Transgenic mice were then generated by the microinjection of DNA into fertilized eggs. The injected eggs were then transferred into the oviducts of pseudopregnant foster females. The genotypes of the mice or embryos were identified by PCR using isolated genomic DNA from the tail or yolk-sac. The primers used were as follows:

*RFP-F2* and *N3-3U-R1* for *BAC-Nanos3-mRFP(Nos3-3′UTR)* and *BAC-ΔNos3-mRFP(Nos3-3′ UTR)*;

*mRFP-F2* and *bghpA-R2* for *BAC-Nanos3-mRFP(BghpA)* and *BAC-ΔNos3-mRFP(BghpA)*. The primer sequences were described in Method S1.

### Immunofluorescence

The mouse embryos and gonads were fixed in 4% PFA for 2 hours at 4°C and washed three times for 5 min each with PBS. After blocking with PBS containing 3% skim milk or 10% FBS for 1 hour at RT, samples were rinsed and incubated overnight with primary antibodies in PBS containing 0.1% TritonX-100 (PBS-Tr) at 4°C or RT. The following day, samples were washed 6 times for 15 min each in PBS-Tr and were incubated for 2 hours at RT with secondary antibodies in PBS-Tr. After the samples had been washed 6 times for 15 min each with PBS-Tr, they were mounted on MAS-coated slide glasses or a glass-bottom dish (Matsunami) and enclosed with PBS by manicure. The samples were then analyzed by confocal laser microscopy (Zeiss).

Primary antibodies were used at the following dilutions: 1∶500 for rabbit anti-NANOS3 [45], 1∶50000 for anti-PGC7 (Sato et al. 2002), 1∶500 for mouse anti-Oct3/4 (C-10) (Santa Cruz sc-5279) and 1∶8000 for rat TRA98 [46]. Secondary antibodies were all used at a 1∶200 dilution (Alexa-488 conjugated donkey anti-rabbit IgG, Alexa-488 conjugated donkey anti-mouse IgG, Alexa-488 conjugated donkey anti-rat IgG, Alexa-594 conjugated donkey anti-mouse IgG and Alexa-594 conjugated donkey anti-rat IgG).

### Evaluation of mRFP Intensity

Samples were fixed in 4%PFA and immunostained with anti-Oct4 and Alexa-488 conjugated donkey anti-mouse IgG to determine germ cells. Then the images were taken using confocal laser microscopy (Zeiss). mRFP intensity in the immunostained embryo did not show significant difference from the unfixed embryo (data not shown). The mRFP intensity of each cell was measured using imageJ software (NIH, Bethesda, MD) and the mean gray values were regarded as the intensity of cell. Three embryos for each stage were examined. All data were normalized using the intrinsic background intensity of wild type embryos at each embryonic stage. The intensity of the E7.5 PGCs was assigned a value of 10 and the data were plotted accordingly.

### Quantitative RT-PCR

Total RNAs were prepared with RNeasy (Qiagen) and used for reverse transcription by Super script III (Invitrogen). Quantitative RT-PCR was performed on the Mini Opticon Real-Time PCR System (Bio-RAD) using SYBR Premix Ex Taq (Takara). Samples were prepared as a pool of cDNA derived from 2–3 pieces of embryos, 4–8 gonads or 4–6 kidneys and each sample was analyzed in triplicate. mRNA levels were calculated with an absolute quantification method and normalized by the amount of *G3PDH* for each sample. The primers used were as follows: *mNos3-F2* and *N3-cod-R1* for *Nanos3* mRNA, *RFP-F2* and *RFP-R2* for mRFP mRNA (including those fused with *Nanos3*), *G3PDH-F* and *G3PDH-R* for *G3pdh* mRNA. The primer sequences were described in Method S1.

## Supporting Information

Figure S1 Both *BAC-Nanos3-mRFP(Nos3-3′UTR)* and *BAC-Nanos3-mRFP(BghpA)* transgenes rescue defects of *Nanos3^−/−^*. HE-stained sections of adult testes (A–D) and ovary (E–H) derived from *Nanos3^+/−^* (A), *Nanos3^+/+^* (E), *Nanos3^−/−^* (B and F), *Nanos3^+/−^* harboring *BAC-Nanos3-mRFP(Nos3-3′UTR)* (C), *Nanos3^+/−^* harboring *BAC-Nanos3-mRFP(Nos3-3′UTR)* (G) and *Nanos3^−/−^* harboring *BAC-Nanos3-mRFP(Nos3-3′UTR)* (D and H) are shown. Scale bar indicates 250 µm. Immunofluorescence images of E14.5 male (I–L) and female (M–P) gonads derived from *Nanos3^+/−^* (I and M), *Nanos3^−/−^* (J and N), *Nanos3^+/−^* harboring *BAC-Nanos3-mRFP(BghpA)* (K and O) and *Nanos3^−/−^* harboring *BAC-Nanos3-mRFP(BghpA)* (L and P) are shown. Masenta represents germ cells (TRA98) and blue represents DNA (DAPI). Scale bar indicates 100 µm. Although *Nanos3^−/−^* had no germ cell, *Nanos3^−/−^* harboring *BAC-Nanos3-mRFP(Nos3-3′UTR)* or harboring *BAC-Nanos3-mRFP(BghpA)* had many germ cells.
(10.04 MB TIF)

Figure S2 *Nanos3-mRFP* protein showed cytoplasmic localizaion in germ cells as well as *Nanos3* protein. Confocal images of embryos of the wild-type at E7.5 (A and B) and E9.5 (C, C′, D and D′). Panels (A–D) show immunostaining with anti-mRFP antibody and the merged images with immunostaining for the germ cell marker anti-OCT3/4 antibody are shown in (C′–D′). Scale bar indicates 100 µm.
(2.28 MB TIF)

Figure S3 3′UTR of other germ cell specific genes was not sufficient for establishing the germ cell-specific expression pattern. The fluorescence images of male embryos derived from *CAG-mRFP(Nos2-3′UTR)* (A), *CAG-mRFP(Stella-3′UTR)* (B) and *CAG-mRFP(TubulinB1-3′UTR)* at E13.5 male (A–B) or female (C). Top images represent the abdomens of embryos harboring transgene (Tg^+^), whereas bottom images represent those harboring no transgene (Tg^−^). Broken gray lines indicate gonads.
(2.28 MB TIF)

Methods S1 Supplementary methods.
(0.03 MB DOC)
