# Supplementary figures and images for: The Nanos3-3′UTR Is Required for Germ Cell Specific NANOS3 Expression in Mouse Embryos
Source: PLoS One. 2010 Feb 18;5(2):e9300. doi: 10.1371/journal.pone.0009300 (PMC2823788; doi:10.1371/journal.pone.0009300)

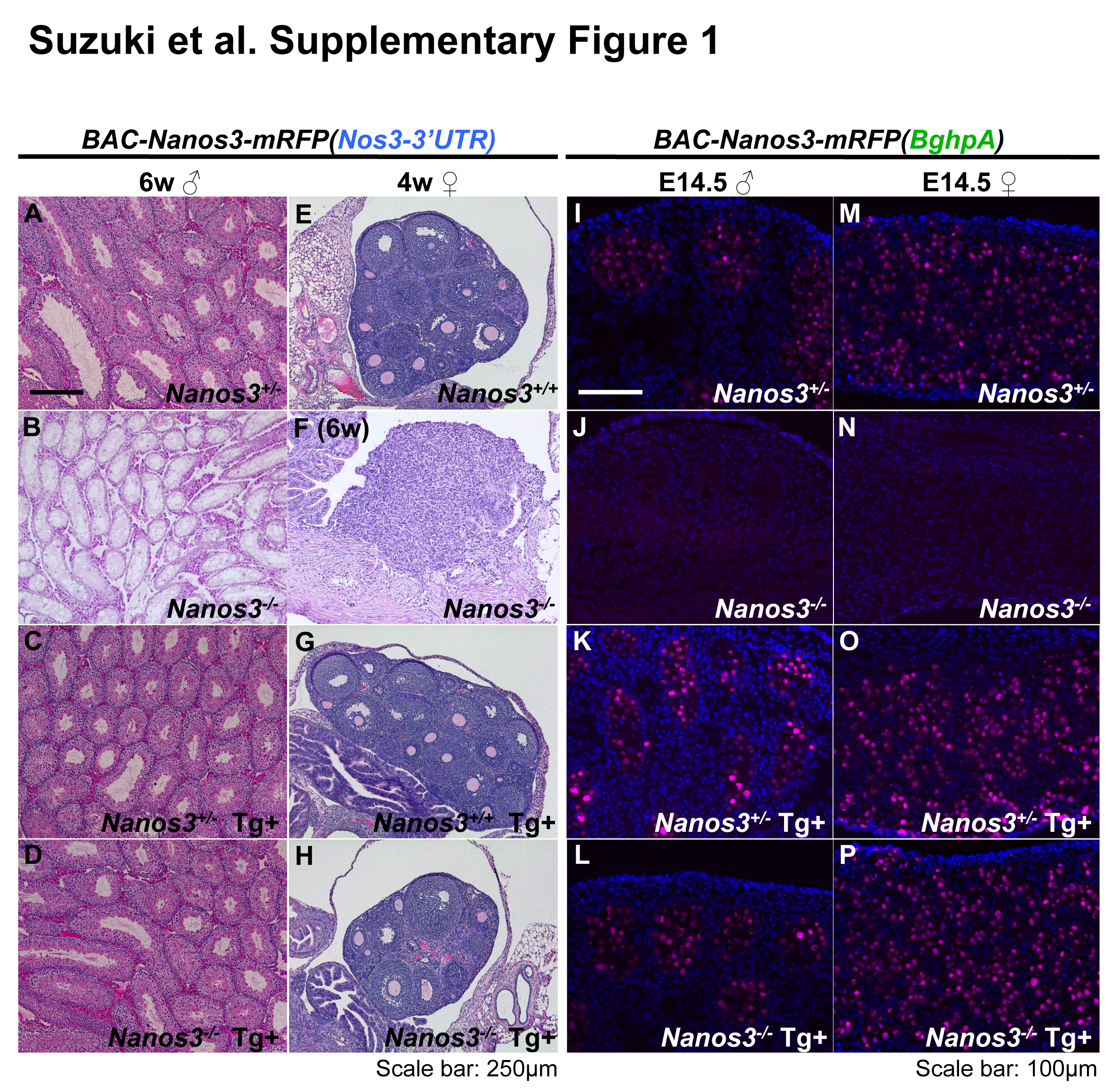

Supplement: Figure S1 — Both BAC-Nanos3-mRFP(Nos3-3′UTR) and BAC-Nanos3-mRFP(BghpA) transgenes rescue defects of Nanos3−/−. HE-stained sections of adult testes (A–D) and ovary (E–H) derived from Nanos3+/− (A), Nanos3+/+ (E), Nanos3−/− (B and F), Nanos3+/− harboring BAC-Nanos3-mRFP(Nos3-3′UTR) (C), Nanos3+/− harboring BAC-Nanos3-mRFP(Nos3-3′UTR) (G) and Nanos3−/− harboring BAC-Nanos3-mRFP(Nos3-3′UTR) (D and H) are shown. Scale bar indicates 250 µm. Immunofluorescence images of E14.5 male (I–L) and female (M–P) gonads derived from Nanos3+/− (I and M), Nanos3−/− (J and N), Nanos3+/− harboring BAC-Nanos3-mRFP(BghpA) (K and O) and Nanos3−/− harboring BAC-Nanos3-mRFP(BghpA) (L and P) are shown. Masenta represents germ cells (TRA98) and blue represents DNA (DAPI). Scale bar indicates 100 µm. Although Nanos3−/− had no germ cell, Nanos3−/− harboring BAC-Nanos3-mRFP(Nos3-3′UTR) or harboring BAC-Nanos3-mRFP(BghpA) had many germ cells. (10.04 MB TIF) [file pone.0009300.s001.tif]

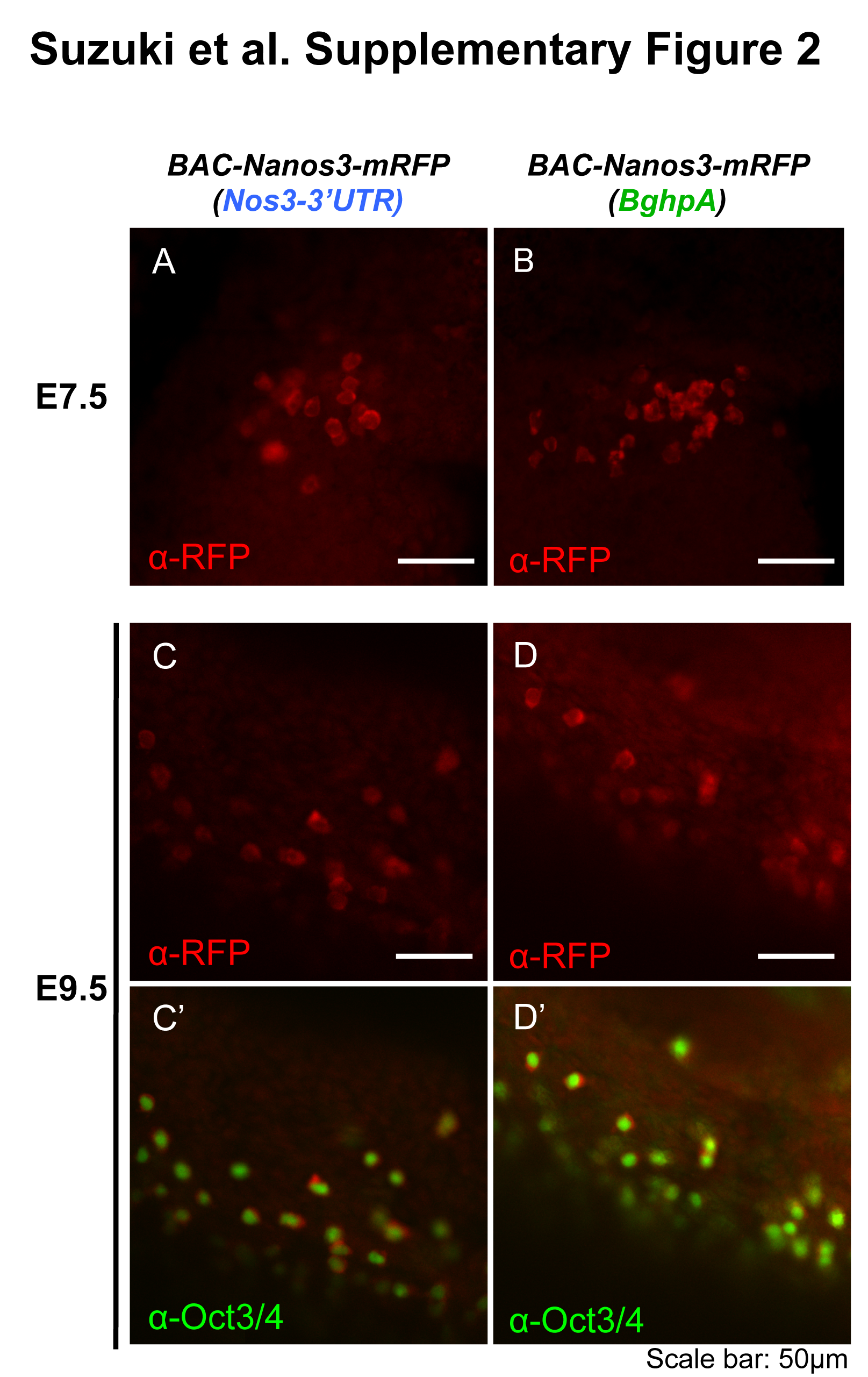

Supplement: Figure S2 — Nanos3-mRFP protein showed cytoplasmic localizaion in germ cells as well as Nanos3 protein. Confocal images of embryos of the wild-type at E7.5 (A and B) and E9.5 (C, C′, D and D′). Panels (A–D) show immunostaining with anti-mRFP antibody and the merged images with immunostaining for the germ cell marker anti-OCT3/4 antibody are shown in (C′–D′). Scale bar indicates 100 µm. (2.28 MB TIF) [file pone.0009300.s002.tif]

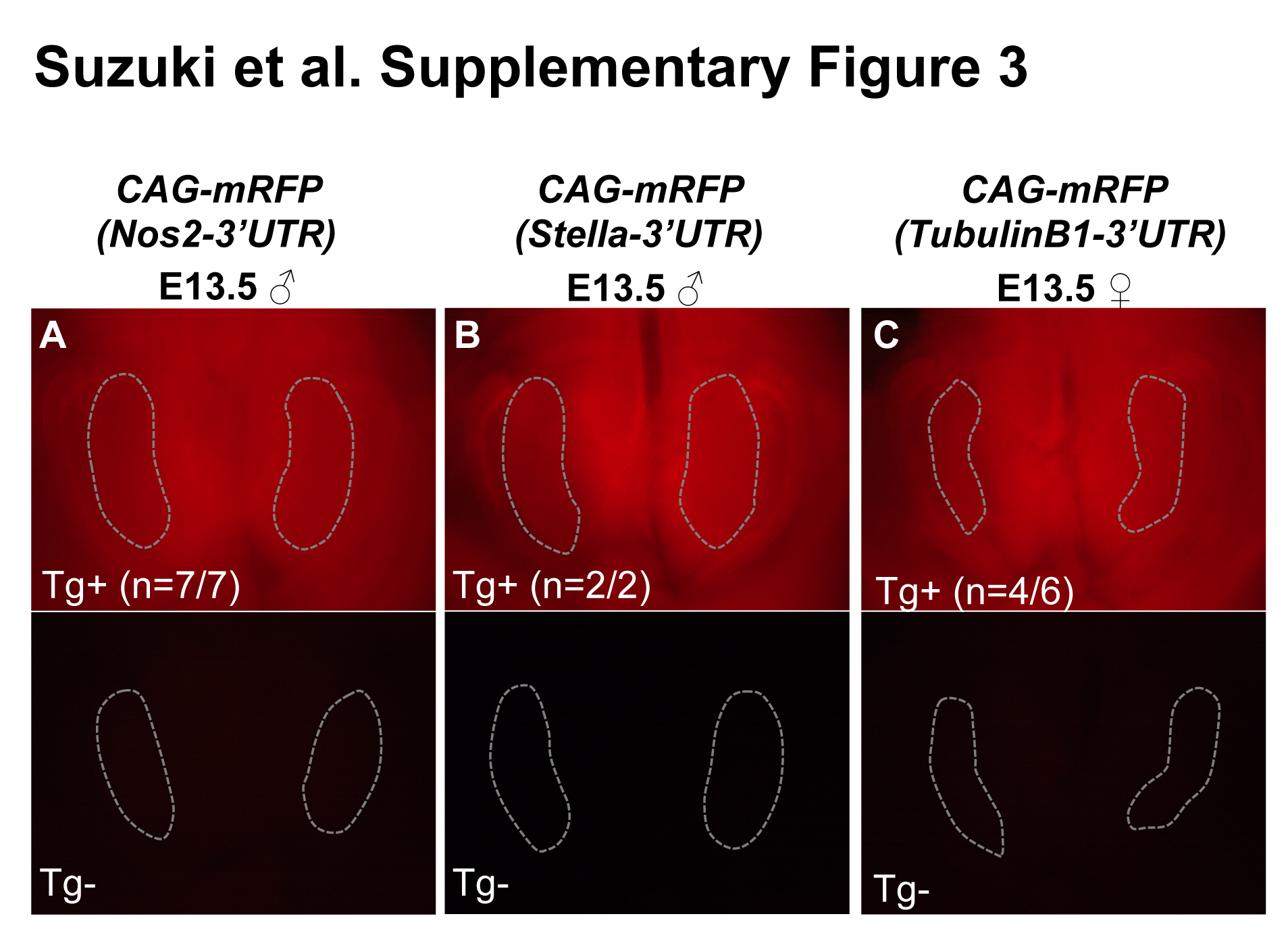

Supplement: Figure S3 — 3′UTR of other germ cell specific genes was not sufficient for establishing the germ cell-specific expression pattern. The fluorescence images of male embryos derived from CAG-mRFP(Nos2-3′UTR) (A), CAG-mRFP(Stella-3′UTR) (B) and CAG-mRFP(TubulinB1-3′UTR) at E13.5 male (A–B) or female (C). Top images represent the abdomens of embryos harboring transgene (Tg+), whereas bottom images represent those harboring no transgene (Tg−). Broken gray lines indicate gonads. (2.28 MB TIF) [file pone.0009300.s003.tif]
