# Supplementary material for: The Nanos3-3′UTR Is Required for Germ Cell Specific NANOS3 Expression in Mouse Embryos
Source: PLoS One. 2010 Feb 18;5(2):e9300. doi: 10.1371/journal.pone.0009300 (PMC2823788; doi:10.1371/journal.pone.0009300)
Supplement: Methods S1 — Supplementary methods. (0.03 MB DOC) [file pone.0009300.s004.doc]

***Histology and Immunofluorescence***

For histology, testes and ovaries were fixed in Bouin’s solution and embedded in paraffin. For hematoxylin and eosin staining, sections (6μm) were deparaffinized, dehydrated, and stained with Mayer’s Hematoxylin (Wako) followed by staining with Eosin Y (Merck).

For Immunofluorescence, the mouse gonads were embedded in OCT compound (Tissue Tek) without fixation. The frozen sections (8μm) were fixed in 4%PFA for 10min at 4°C and washed in PBS three times. After blocking with PBS containing 3% skim milk for 1 hour at RT, sections were rinsed and incubated overnight with primary antibody in PBS at 4°C. The following day, sections were washed 3 times in PBS and were incubated for 1 hours at RT with secondary antibody in PBS. These sections were then counterstained with DAPI (Sigma D-9542) and mounted with Gel/Mount (Biomeda). Primary antibodies were used at 1:5000 dilution for rat TRA98. Secondary antibody Alexa-594 conjugated donkey anti-rat IgG was used at a 1:200 dilution.

***Cloning of 3’UTR sequences***

*Nanos3-*3’UTR was cloned by PCR on DNA template prepared from the tail of C57BL6/J strain mouse. The primers used are as follows:

*Nos2-3U-SalI-F* and *Nos2-3U-HindIII-R* for *Nanos2*-3’UTR;

*Stella-3U-SalI-F* and *Stella-3U-HindIII-R* for *Stella/PGC7*-3’UTR;

*TubB1-3U-SalI-F* and *TubB1-3U-HindIII-R* for *Tubulin B1*-3’UTR.

***Primer Sequences***

*BAC-Nanos3/mRFP-F*: (5’-GGAAGCCCCCTGGACCTTCACCCTCGGCCTGCTGTCCCTCCACTACGGCCGCCTCCTCCGAGGACGTCAT-3’),

*BAC-Nanos3(fusion-3'UTR)-R*: (5’-CTGCCCGCCAGATTCATACCACCTTGTCCCAAGACATGCTCCACTCCGTGTAGGCTGGAGCTGCTTC-3’)

*BAC-Nanos3(ATG)/mRFP-F*: (5’-GGAAGTTGGAGCCAGGTTGGGTTTCTTCTCTGCTCCTCTGCCCAGCCATGGCCTCCTCCGAGGACGTCAT-3’)

*N3-IN-F1*: (5’ – AACAGGAACACCTCCCTAGT-3’)

*N3-LA-KR1*: (5’- TGGTAGCAGCATCTCAGG-3’)

*N3-stop-SalI-F1*: (5’-TACGGTCGACGAGCTTGGAGTGGGGA-3’)

*N3-3'U-HindIII-R1*: (5’-CAAGAAAGCTTCCACTCCTTAGCATTTA-3’)

*Stella-3U-SalI-F*: (5’-GTCGACGAGCTTACATTGTACGCT-3’)

*Stella-3U-HindIII-R*: (5’-AAGCTTAACAAAAATGCTTTTATTAC-3’)

*TUBB1-3U-SalI-F*: (5’-ATCGTCGACGCCAAGAGGAAAAGCTCT-3’)

*TUBB1-3U-HindIII-R*: (5’-GGCAAGCTTGAAAGCAGATATTTGTAT-3’)

*RFP-F2H*: (5’-CGAGATCAAGATGAGGCTG-3’)

*N3-3U-R1*: (5’-GGATGTTGAGGCAACACC-3’)

*bghpA-R2*: (5’-TTCCGCCTCAGAAGCCATAGA-3’)

*mNos3-F2*: (5’-TCCCGTGCCATCTATCAG-3’)

*N3-cod-R1*: (5’-GGCTTCCTGCCACTTTTG-3’)

*RFP-F2*: (5’-AAGCTGAAGGTGACCAAGGG-3’)

*RFP-R2*: (5’-GTAGGTGGTCTTGACCTCGG-3’)

*G3PDH-F*: (5’-ACCACAGTCCATGCCATCAC-3’)

*G3PDH-R*: (5’-TCCACCACCCTGTTGCTGTA-3’)
